# Indicators for Tracking European Vulnerabilities to the Risks of Infectious Disease Transmission due to Climate Change 

**DOI:** 10.3390/ijerph110202218

**Published:** 2014-02-21

**Authors:** Jonathan E. Suk, Kristie L. Ebi, David Vose, Willy Wint, Neil Alexander, Koen Mintiens, Jan C. Semenza

**Affiliations:** 1 European Centre for Disease Prevention and Control, Tomtebodavägen 11A, Stockholm 17183, Sweden; E-Mail: jonathan.suk@ecdc.europa.eu; 2 ClimAdapt LLC, Los Altos, CA 94022, USA; E-Mail: krisebi@essllc.org; 3 Vose Software, Franklin Rooseveltlaan 348, Ghent 9000, Belgium; E-Mail: david@vosesoftware.com; 4 Environmental Research Group Oxford, Department of Zoology, University of Oxford, South Parks Road, Oxford, OX1 3PS, UK; E-Mails: william.wint@zoo.ox.ac.uk (W.W.); neil.alexander@zoo.ox.ac.uk (N.A.); 5 Avia-GIS, Risschotlei 33, Zoersel 2280, Belgium; E-Mail: koen.mintiens@boerenbond.be

**Keywords:** infectious disease, public health, preparedness, climate change, adaptation, adaptive capacity, vulnerability, horizon scanning, Europe

## Abstract

A wide range of infectious diseases may change their geographic range, seasonality and incidence due to climate change, but there is limited research exploring health vulnerabilities to climate change. In order to address this gap, pan-European vulnerability indices were developed for 2035 and 2055, based upon the definition vulnerability = impact/adaptive capacity. Future impacts were projected based upon changes in temperature and precipitation patterns, whilst adaptive capacity was developed from the results of a previous pan-European study. The results were plotted via ArcGIS^TM^ to EU regional (NUTS2) levels for 2035 and 2055 and ranked according to quintiles. The models demonstrate regional variations with respect to projected climate-related infectious disease challenges that they will face, and with respect to projected vulnerabilities after accounting for regional adaptive capacities. Regions with higher adaptive capacities, such as in Scandinavia and central Europe, will likely be better able to offset any climate change impacts and are thus generally less vulnerable than areas with lower adaptive capacities. The indices developed here provide public health planners with information to guide prioritisation of activities aimed at strengthening regional preparedness for the health impacts of climate change. There are, however, many limitations and uncertainties when modeling health vulnerabilities. To further advance the field, the importance of variables such as coping capacity and governance should be better accounted for, and there is the need to systematically collect and analyse the interlinkages between the numerous and ever-expanding environmental, socioeconomic, demographic and epidemiologic datasets so as to promote the public health capacity to detect, forecast, and prepare for the health threats due to climate change.

## 1. Introduction

A fairly wide range of infectious disease may change their geographic range, seasonality, incidence or prevalence with climate and environmental change [1]. For example, the tick species *Ixinus ricinus*, an important vector for tick-borne encephalitis and lyme borreliosis, has spread into higher latitudes [2] due in part to warmer temperatures. In Europe the climate is increasingly suitable for the mosquito species *Aedes albopictus*, also due in part to warmer temperatures [3,4]. In southeast Europe, high summer temperatures facilitated the transmission of West Nile fever in 2010 [5]; and possible outbreaks of food- and water-borne diseases such as salmonella, cryptosporidium, VTEC, and campylobacter, in part due to changing temperature and precipitation patterns [6]. 

Monitoring and evaluation are essential components of public health and health care programs to manage the projected risks of infectious diseases under a changing climate [7]. Current and projected health risks due to climate change result from the hazards associated with climate change interacting with existing vulnerabilities and with the ability of individuals and communities to cope with the risks [8,9]. Indicators of vulnerability therefore need to consider who is exposed at present and in the future to particular hazards arising from climate change, including changes in the mean and variability of weather values; the susceptibility of exposed individuals and communities; and the capacity of those exposed (including the public health and health care institutions whose mandate is to care for those exposed) to avoid, prepare for, cope with, and recover from impacts [10]. Public health conceptual frameworks for the health risks of climate change [11,12] need to be updated to include all these dimensions of risk. 

European national-level expert opinion has been previously used to identify vulnerabilities to the risks posed by climate change on infectious disease transmission [13]. In this study, we explore the feasibility of developing a quantitative indicator to compare regional vulnerabilities to the health effects of climate change, and we evaluate the benefits and limitations of such an approach.

## 2. Methods

### 2.1. Definitions of Vulnerability and Adaptive Capacity

An essential starting point for this study was to identify a working definition of vulnerability, which can be conceptually defined as a predisposition or propensity to be adversely affected [14]. The vulnerability of a population or a location is the summation of all the risk and protective factors that collectively determine whether a subpopulation or region experiences adverse health outcomes [15]. The vulnerability of populations, similar to infectious disease risks, varies across spatial and temporal scales in response to changes in economic development, social capital, the demographic structure of a population (such as the proportion of elderly people in a population or the degree of urbanisation), trade and travel patterns, the prevalence of pre-existing medical conditions, acquired factors (such as immunity), and genetic and other factors [10,15,16,17,18].

The ability to prepare for and cope with the risks of climate change is a function of the status of the public health and health care infrastructure, such as the quality of surveillance and control programs, social capital, distribution of resources, treatment costs, ability to adapt, education levels, and so on. By mediating risk and/or differentially affecting the ability to prepare for or respond to hazards, socioeconomic factors play a critical role in determining both vulnerability as well as the risk of disease [19]. 

Adaptive capacity has been defined by IPCC as “the ability or potential of a system to respond successfully to climate variability and change” [20]. There is no *a priori* agreement about what should be the most suitable components of an index for adaptive capacity for the impacts of climate change on infectious disease transmission [21]. Few health-related studies have considered critical components of adaptive capacity. One study assessed decadal aggregated mortality from climate-related disasters [22]. Eleven indicators had a strong association with decadal aggregated mortality, which were grouped into four categories: health status; the efficacy of health care systems; governance; and education. Another study used a conjoint choice survey of public health and climate change experts at professional meetings to assess determinants of the capacity of countries to address the health risks of climate change; this information was then used to construct an index of adaptive capacity [23]. The respondents viewed per capita income, inequality in the distribution of income, universal health care coverage, and high access to information as important determinants. 

An important challenge is to convert the desired variables into an index that can be operationalised. One approach for doing so was described by the European Observation Network, Territorial Development and Cohesion (ESPON) for modelling vulnerability [24]. ESPON combines estimates of the risk that a population may face together with adaptive capacity to develop a more operational (but somewhat less holistic) definition of vulnerability than the one described at the beginning of this chapter:

*Vulnerability = Impact / Adaptive Capacity*

The assumption underpinning the reciprocal relationship is that the higher the adaptive capacity of a region, the lower its *Vulnerability*. This definition implies that it is necessary to project the impact of climate change on infectious disease transmission as well as adaptive capacity in order to assess vulnerability. 

**Figure 1:**
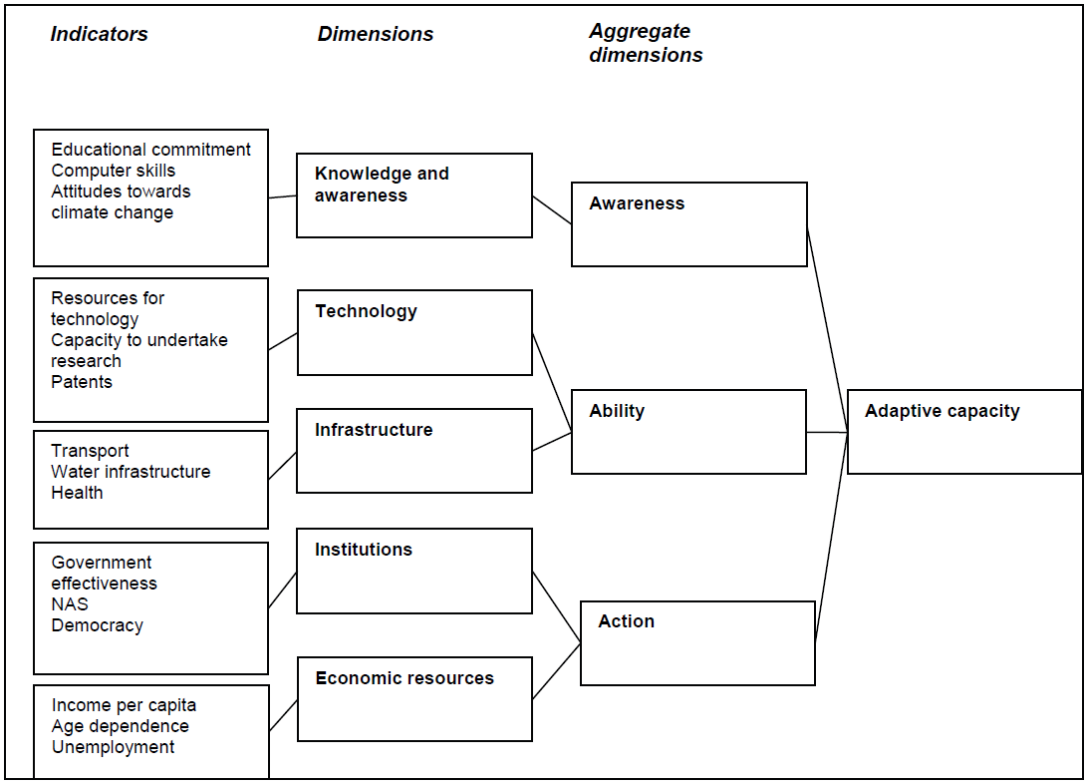
Composition of the adaptive capacity developed by ESPON (^©^ ESPON 2013).

The ESPON study nevertheless provides a comprehensive and consistent EU-wide attempt to map adaptive capacity, with resolution at the NUTS 3 level [24]. Many of the variables in the studies cited earlier fit into the ESPON conceptual framework (Table S1). Moreover, ESPON systematically attempted to assess societal-wide adaptive capacity through a range of indicators for five dimensions (knowledge and awareness, technology, infrastructure, institutions and economic resources) that could be further aggregated into three key dimensions (awareness, ability, action) influencing adaptive capacity (Figure 1). ESPON used a Delphi elicitation process to combine and weight the factors shown in Figure 1 to derive an adaptive capacity index at the European sub-regional (NUTS 3) level according to the Eurostat nomenclature of territorial units for statistics [25]. The aggregated weightings assigned to the five factors were the physical (weight 0.19), environmental (0.31), social (0.16), economic (0.24) and cultural (0.1) impacts of climate change. The inputs for each group are identified in Figure 1. For the purposes of the analysis in this study, we averaged the values to achieve an adaptive capacity at the regional (subnational) NUTS 2 level of resolution.

### 2.2. Study Parameters

The objective was to develop a regional-level, European-wide index of vulnerability to the infectious disease risks of climate change. Rather than assessing present-day vulnerabilities, the index was developed for the years 2035 and 2055. The study area consisted of European Union Member States at the regional (*i.e.*, subnational) level (NUTS 2). Importantly, the vulnerability index is general in nature, rather than being disease-specific, meaning that the creation of the index did not involve considering in detail the myriad transmission pathways relevant to the full spectrum of infectious diseases in Europe. The goal was to create a general vulnerability index that could then be combined with knowledge of estimated risks for particular infectious diseases to identify specific indicators for monitoring key risks associated with climate change. 

### 2.3. Data Inputs, Transformation and Analysis

Based on the ESPON definition of vulnerability, the next step was to decide on the data inputs for its key components, impact and adaptive capacity.

### 2.4. Impacts

Climate-sensitive diseases can be fairly reasonably expected to undergo significant and/or unanticipated shifts in geographic range or seasonality, as well as altered transmission patterns, where climatic changes are the greatest. The most reliable climate projections are for temperature and precipitation, and although the frequency of extreme events, such as flooding and heavy rainfall events, are also relevant to infectious diseases, reliable and high-resolution projections for these variables are not as robust [14]. Thus, temperature and precipitation were selected as the two input variables for the impact side of the equation.

Rather than creating outputs from individual climate models and scenarios, this analysis adopted an ensemble approach by performing a single analysis using a climate model ensemble that combines a set of independent model outputs and scenarios together with the resulting standard deviations. Thus, projection of European-wide monthly means for daily temperature (T_min_ and T_max_ in degrees C) and daily precipitation in mm for 2035 and 2055 were available at 10 km resolution from the FutureClim datasets produced using a method described elsewhere [26]. A multi model ensemble based upon three emission scenarios (A1b, A2, B1) [27] and four earth system models (ECHAM5, MIROC3, CNRM and CSIRO3), downscaled to 1km resolution using the same techniques commissioned by the Environmental Research Group Oxford and made available to this project and to members of the EDENext consortium.

Two ways of developing the adaptive capacity index were explored. First, a core set of desired variables relevant for infectious disease control was selected. Identified indicators were selected based upon data availability and validity as proxy values for awareness, ability, and action (Table 1). It was decided to base adaptive capacity on present-day data rather than future projections because it is much harder to obtain future projections of relevant socioeconomic data than it is for climate data: the great uncertainty inherent in any socioeconomic projections would contribute to the multiplication of overall model uncertainties [28]. Furthermore, major changes to the European public health landscape are more likely to be the consequence of changing political paradigms or other extrinsic factors that can neither be predicted nor accurately incorporated into models.

The data in Table 1 were evaluated for their usefulness as data inputs. One problematic factor was that certain potential datasets did not adequately account for regional variability and differential healthcare system structures. For example, the number of hospital beds per 100,000 inhabitants (at NUTS2 level) was considered but during the ten years between 1998 and 2008, the number of hospital beds per 100,000 inhabitants fell in every Member State, except Malta (where the main general hospital was reconstructed) whilst, simultaneously, general health levels increased. The largest reductions in the availability of hospital beds were recorded in the three Baltic Member States and in Bulgaria. The reduction in hospital bed numbers may reflect, among others, economic constraints, increased efficiency through the use of technical resources (for example, imaging equipment), a general shift from inpatient to outpatient operations, and shorter periods spent in hospital following an operation [25]. In other words, the number of hospital beds can be inversely correlated with the level of healthcare, and thus it is not possible from the available data to tease out the benefit of the availability of hospital capacity in terms of its healthcare value.

**Table 1:** Sample datasets for consideration as components of adaptive capacity.

Component of vulnerability	Desired variable	Proxy variable	Spatial resolution
Adaptive capacity: awareness	Individuals with limited understanding of aetiology of infectious disease	Education: literacy rate	NUTS2
Adaptive capacity: ability	Status of health care	Health care personnel per 100,000 population	NUTS2
		Life expectancy at birth	
		Under 5 mortality rate	
			NUTS2
	Individuals susceptible to infectious disease	% population <5 years of age % population >65 years of age	NUTS2
Adaptive capacity: action	Income	Gross Domestic Product (GDP) per capita	NUTS 2
	Funding of healthcare system	Projected changes in health care spending as:	
		% GDP	COUNTRY
		% total health expenditure	COUNTRY
		% of total government spending	COUNTRY

Similarly, the number of medical staff or physicians is a poor reflection of medical care as access to modern drugs, treatment techniques, surveillance systems and equipment all play a major role. In another example, infant mortality rates have reduced and life expectancies have increased across the EU27 between 1998 and 2008 [25]. Life expectancy at birth is a projection based on models and predictions (see, for example, improvements for Eastern European countries). Under-5 mortality is monitored, but improvements are more expected to be due to medical, technical and infrastructure than to any climatic changes.

Such limitations demonstrate the types of challenges in identifying proxy variables for adaptive capacity directly related to the health sector. Working with broader socioeconomic measures, meanwhile, weakens analytical sensitivity. Despite this, the decision was made to interrogate adaptive capacity broadly, without relying too heavily on overly narrowly focused datasets. Thus, the decision was made to deploy the ESPON adaptive capacity dataset (^©^ ESPON Database) [24]. The final datasets incorporated in the vulnerability index are described in Table 2.

**Table 2:** Datasets used in the final vulnerability index.

Component of Adaptive Capacity	Desired Variable	Proxy Variable	Spatial Resolution
Hazard	Climate variability and change in 2035 and 2055	Average temperature Average precipitation	NUTS2 NUTS2
Adaptive capacity: overall	Adaptive capacity index for Europe (general)	ESPON adaptive capacity index	NUTS 2

### 2.5. Development of the Vulnerability Index

The vulnerability index (*VI)* for an EU Member State region *r* in year *t* was defined as:

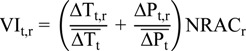

where:

- NRAC_r_ is the reciprocal of the adaptive capacity as described by ESPON [24] normalized to average to 1 over all regions;
- ∆T_t_ = |*MinT_t_* − *MinT_now_*| + |*MaxT_t_* − *MaxT_now_*|
- ∆*P_t_* = |*MinP_t_* − *MinP_now_*| + |*MaxP_t_* − *MaxP_now_*|
- *MinP, MaxP, MinT,* and *MaxT* are the monthly projected precipitation and temperature extremes respectively; and
- ∆T_t_ and ∆*P_t_* are the ∆T_t_ and ∆*P_t_* variables averaged over all values at NUTS2 level within the EU27 in year *t*.

Note that the index considers the extremes as a change *relative* to the average ∆*T_t_* or ∆*P_t_* that is projected, *i.e.*,:

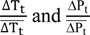



The choice of using ratios of differences was made to avoid two problems. An alternative might have been to develop the index:

VI_Alt1,t_ = (T_t_ + kP_t_)NRAC_r_

where *k* is some constant to weight the relative importance of the two physical properties which would have to be subjectively assigned. Moreover, the index should work independent of the scale of the variables. The Celsius scale for temperature is arbitrary and 0 °C is just one point on a continuum, whereas zero is the absolute minimum for precipitation. The true ‘zero’ for temperature is at −273 °C, so variations in the range of roughly −20 °C to +40 °C would register as a smaller difference to *T_t_* in the Kelvin scale. 

Another option could have been:

VI_Alt2,t_ = (∆T_t_ + k∆P_t_)NRAC_r_

but, again, this would require assigning an arbitrary weighting (the factor *k*) between the two physical properties.

An implied assumption is that within a Member State all members of the population have equal access to the same level of healthcare. This, of course, is not true but is a necessary simplifying assumption. Note that this index does not take into account of the number of people residing in a particular NUTS2 region.

### 2.6. Assessment of Indices

To assess the key drivers of the impact indices, a Spearman rank correlation test was run. This non-parametric test was used because it is has no implied assumption about the correlation structure between variables. The scale runs from −1 to 1, with 0 in the middle equating to no rank correlation. The larger the absolute value of the rank correlation, the greater the influence that an input variable has on the rank of the output.

### 2.7. Mapping Approach

The index results for the impact and vulnerability indices were plotted via ArcGIS^TM^ to EU NUTS2 levels for 2035 and 2055. Impact indices and overall vulnerability indices were plotted for both time periods to assist visualisation of the role of adaptive capacity. The results are presented according to a relative ranking based on quintiles, which enabled a clearer visual discrimination between classes so as to facilitate a relative comparison between regions. 

## 3. Results

For all maps, red indicates the quintile with the highest projected impact and vulnerabilities, and green the quintile with the lowest projected impacts and overall vulnerabilities. All indices are thus relative, not absolute. 

**Figure 2:**
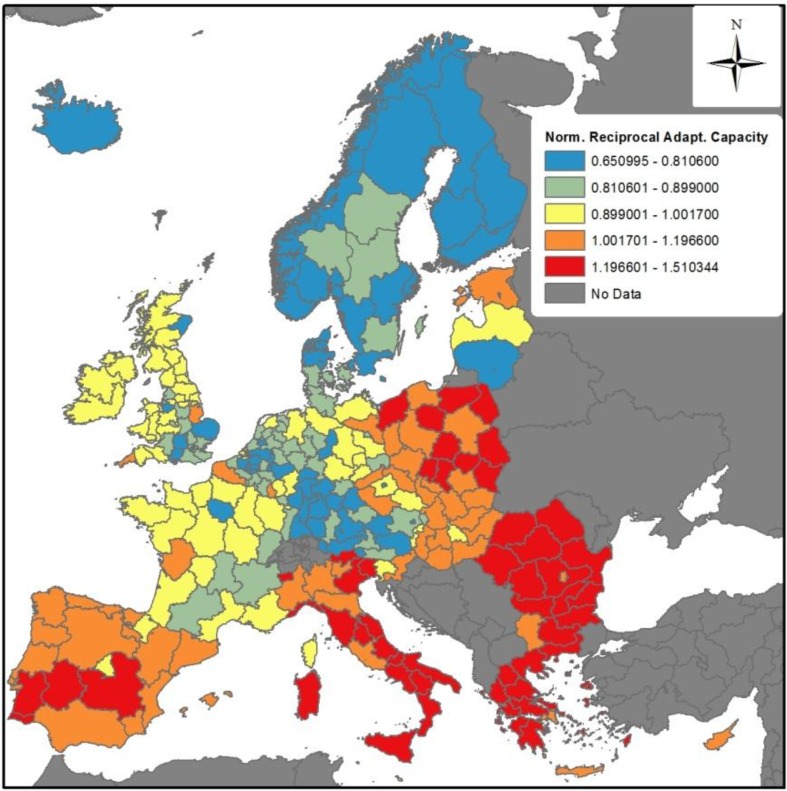
Normalised reciprocal of ESPON Adaptive Capacity index.

The normalised reciprocal of the adaptive capacity index, derived from EPSON and ranked in quintiles, is presented in Figure 2. Given the definition of vulnerability used in this study (vulnerability = impact/adaptive capacity), this index represents the baseline for this study, for no future impacts are projected in this model. The regional-level adaptive capacity in Figure 2 shows that the highest adaptive capacities (and thus lowest baseline vulnerabilities) are concentrated in Scandinavia, south-east England, and central Europe. Conversely, the lowest adaptive capacities (and thus highest baseline vulnerabilities) are concentrated in southern and eastern Europe.

**Figure 3:**
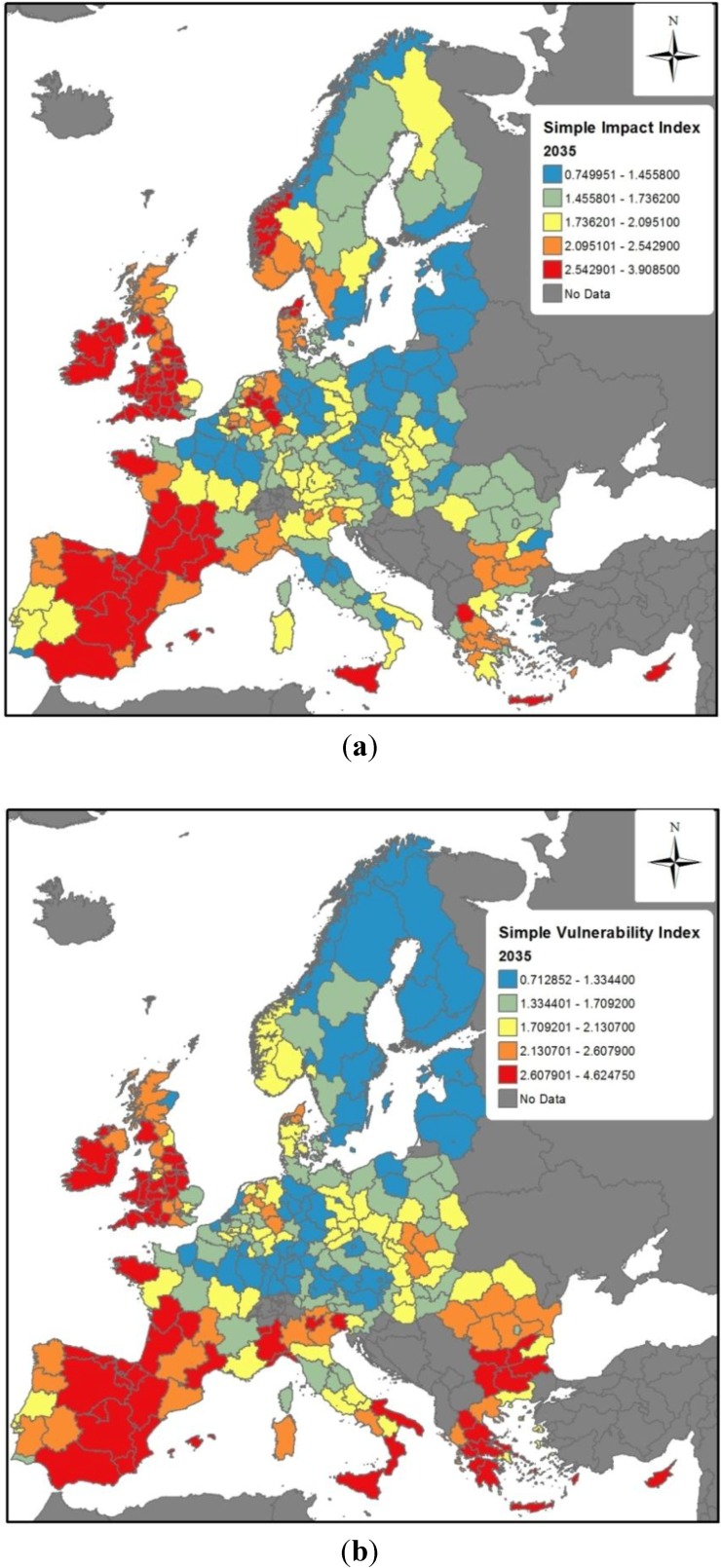
Vulnerability Index projected for 2035: (**a**) Impact Index; (**b**) Vulnerability Index.

**Figure 4:**
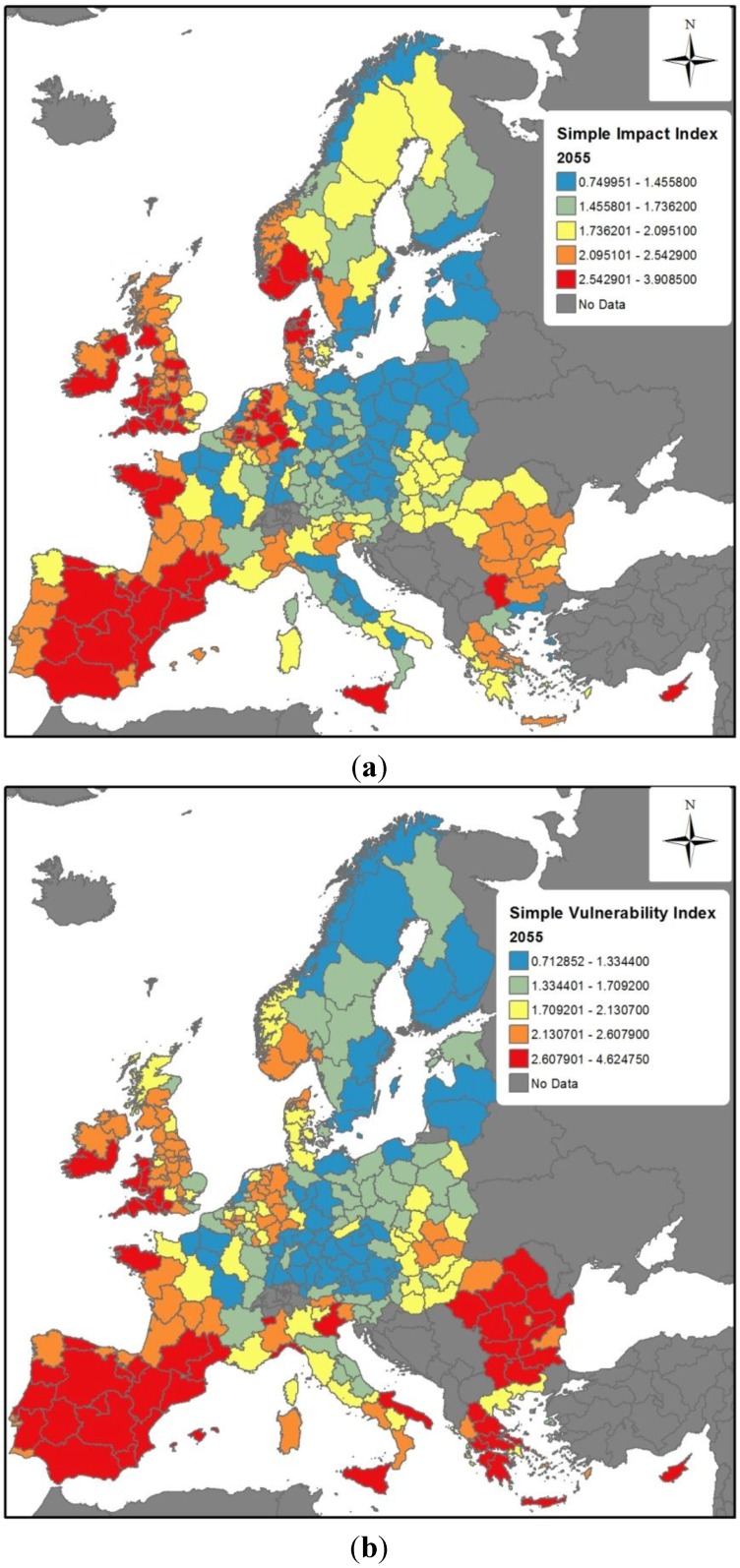
Vulnerability Index projected for 2055. (**a**) Impact Index; (**b**) Vulnerability Index.

The final impact and vulnerability indices for 2035 and 2055 are presented in Figure 3 and Figure 4, respectively. The top panels for these figures show the relative influence of temperature and precipitation change across Europe (impact indices alone), whereas the bottom panels include the adaptive capacity index from Figure 2 to provide the final vulnerability indices. 

**Figure 5:**
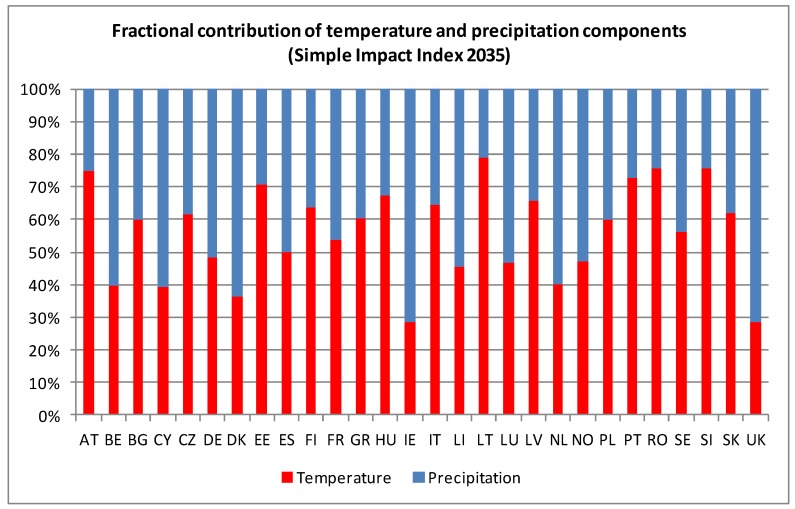
Fractional contribution of the precipitation and temperature components to the value of the Simple Impact Index for 2035 (averaged over each EU Member State).

Notes: AT: Austria. BE: Belgium. BG: Bulgaria. CY: Cyprus. CZ: Czech Republic. DE: Germany. DK: Denmark. EE: Estonia. ES: Spain. FI: Finland. FR: France. GR: Greece. HU: Hungary. IE: Ireland. IT: Italy. LI: Liechtenstein. LT: Lithuania. LU: Luxembourg. LV: Latvia. NL: Netherlands. NO: Norway. PL: Poland. PT: Portugal. RO: Romania. SE: Sweden. SI: Slovenia. SK: Slovakia. UK: United Kingdom.

For the impact indices for 2035 and 2055 (Figure 3a and Figure 4a), regions to the red side of the scale are projected to encounter the largest climate-related infectious disease problems, because they will be the ones facing the largest changes in temperature and/or precipitation, which in turn can be expected to affect the introduction, establishment and transmission of infectious diseases. Both figures present a broad spatial heterogeneity across Europe. The reasons behind differences in projected impacts can differ. Even in regions with higher projected impacts, such as many in Spain and the UK, the reasons can differ. The relative contribution of changing temperature and precipitation affects different countries, as shown in Figure 5, which presents the average fractional contribution that precipitation and temperature have per Member State in the impact indices for 2035. For example, temperature and precipitation contribute equally to the projected impacts in Spain, whereas precipitation accounts for a greater role in the UK impact index (slightly more than 70%). It must furthermore be noted here that the results presented in Figure 5 must be interpreted in the context of absolute values; the large role that precipitation plays in the impact for Cyprus (CY) or Spain (ES), for example, is a significant *decrease* in precipitation, whilst in Ireland (IE) and the UK impacts driven by a substantial *increase* in precipitation. The findings from the Spearman rank correlation (Table 3) furthermore note that precipitation is overall the most influential variable. However, the relative importance of precipitation and temperature on the impact indices is regionally dependent. 

**Table 3:** Rank correlation of input variables with the Simple Impact Index by year.

Rank correlations with Impact Index	2035	2055
|*MinP_t_* − *MinP_now_*|	0.49	0.45
|*MaxP_t_* − *MaxP_now_*|	0.80	0.81
|*MinT_t_* − *MinT_now_*|	0.41	0.40
|*MaxT_t_* − *MaxT_now_*|	−0.03	−0.06

Figure 3b and Figure 4b show the overall vulnerability indices for 2035 and 2055, respectively. In comparison to Figure 3a and Figure 4a, the overall vulnerability indices demonstrate the effect of adaptive capacity in either mitigating or exacerbating vulnerabilities. For both 2035 and 2055, the adaptive capacity of some regions helps to mitigate the impacts from climate change. For example, whereas the Norwegian region including Oslo has projected impacts in high impact quintiles in 2035 and 2055, its adaptive capacity helps to mitigate this somewhat, leading to a somewhat lower vulnerability rankings. Other areas exhibiting similar patterns include southeast England, and numerous regions in Norway, Denmark and Sweden, southern Germany and Austria. Conversely, regions in Romania, Bulgaria, Greece and southern Italy end up in higher vulnerability index quintiles (Figure 3b and Figure 4b) than their respect impact index quintiles (Figure 3a and Figure 4a). This reflects the lower adaptive capacity of these areas. 

It is finally noteworthy that several regions, notably in the Iberian peninsula, the UK and Ireland, and southern France, are projected in the higher quintile ranges in both impact and vulnerability indices. The message here is that regardless of these regions’ specific adaptive capacities, they are not high enough to offset the rather high level of projected climate changes.

## 4. Discussion and Conclusions

It is important to understand the magnitude and pattern of projected climate changes in order to understand how they could impact infectious disease transmission. Yet, without understanding the vulnerabilities and coping capacities of the regions undergoing such changes, it is impossible to develop a comprehensive understanding of the full nature of the threat and the opportunities for interventions to increase resilience. A holistic approach that assesses vulnerabilities in light of both projected impacts and adaptive capacities is essential to strategically embark upon necessary climate change adaptation and public health preparedness measures.

The growing area of vulnerability research offers an intriguing avenue for doing so, for it explicitly draws attention to socioeconomic and political conditions, the susceptibilities of societies and ecological systems to harm, and the ability of these systems to cope with, respond to, and recover from climate-driven events [9]. A key outstanding question is which indicators would be most effective to further the understanding of how climate change might interact with future vulnerability to promote or reduce health risks. Such indicators would ideally account not only for climate change but also the many drivers of infectious disease, which include socioeconomic circumstances, demographic factors, and policy and governance contexts [18,29,30]. There is a large body of research discussing indictors for monitoring temperature, precipitation, and other weather factors as the climate changes, as well as the human and natural systems exposed to those changes. Literature on the social dimensions of vulnerability, including the social processes that affect vulnerabilities, is also rapidly expanding [31]. 

Although there is a robust literature on population susceptibility to infectious disease risks, this literature generally does not consider the risks posed by climate change. For example, where studies addressed the social and environmental dimensions of vulnerabilities related to infectious disease, such as dengue, they consider climate but have yet to address climate change [32,33]. Further, there is very limited research on how these susceptibility factors could change under different development and climate patterns, and there is limited research on indicators of vulnerability and public health preparedness to track whether there is adequate and timely adaptive management to effectively prepare for risks. Where data exists, more evaluation of the sensitivity and utility of data is required [34].

In order to address this gap, in this study the vulnerability models developed (Figure 3b and Figure 4b) consider both the magnitude of climate-driven impacts that a given European region is anticipated to face, as well as the region’s ability to respond—its adaptive capacity as assessed by the current resources (human, financial, infrastructure, *etc.*) that a region could be expected to mobilise so as to reduce impacts. The vulnerability indices for 2035 and 2055 presented here offer one of the first attempts to map pan-European regional vulnerabilities to the impacts of climate change on infectious disease. The indices are necessarily broad-brushed, but provide public health planners with an overview of some European regions that may likely require additional attention to enhance preparedness for climate change. 

The models presented in this study demonstrate that regions projected to undergo the most substantial climate changes but that do not have strong adaptive capacities are particularly vulnerable. In some instances, such as in many regions of south-east Europe, high vulnerabilities are driven by low underlying adaptive capacities. In others, such as in many areas of southern France, the UK, and Ireland, regions may have relatively strong or average levels of adaptive capacity but nonetheless be in the highest vulnerability quintile due to the relatively high influence of projected climate changes. 

There are many limitations to this study. The models have not been validated, which is of course a longstanding challenge for the climate change impacts community. There is no simple solution for validating future-orientated models. One solution could have been “hindcasting”, through which the metrics used in the models would be tested through examining how well they performed over the past several decades. Unfortunately, however, this sort of validation is not possible due to the lack of available, comprehensive and EU-wide datasets that go back so far in time. Another limitation of the study is that it looks broadly at relative differences between European regions in order to create a preliminary prioritisation of regions to address. The alternative could have been to model absolute changes in climate change impacts and/or adaptive capacities, which could be important for specific diseases or other health-related aspects that are not driven by relative climatic changes but rather climate thresholds or “tipping points”. Moving forward, attention should be placed on developing more disease-specific and detailed health indicators of vulnerability, to be able to shed more light on which risk groups are most vulnerable; which susceptibilities are likely to be most important; and the potential of health systems to prepare for, cope with, and recover from the impacts of climate change. With respect to infectious diseases, the development of disease-specific indices could assist more focused adaptation activities, through monitoring data more directly relevant to the disease in question. Such work represents a next phase in this study. 

Some of the broader limitations of this study allude to the critical challenges facing the health sector as it begins to plan and implement options to manage public health vulnerabilities to climate change [35]. One is that although the sector has great experience in programme monitoring and evaluation, it has limited experience anticipating future health challenges under different climate and development pathways. Compared with other sectors, there is much less research from the health sector projecting socioeconomic vulnerabilities to climate change under different development pathways. One key issue is the lack of a consistent and health-relevant definition of vulnerability. The definition of vulnerability used in this study (vulnerability = impact/adaptive capacity) implies that adaptive capacity is one component of vulnerability, yet such an approach is not fully consistent with current understandings of risk, impact, and vulnerability [10]. The definition used in this study implies that vulnerability is similar to “residual impacts”. In other words, it notes the vulnerability remaining after an impact has occurred and all possible actions have been taken to avoid, prepare for, and cope with that impact. This conceptual model is not consistent with the more holistic manner in which public health views vulnerability. 

Thus, although many elements of the ESPON definition of adaptive capacity are similar to those typically used in public health [21], the field could be advanced by identifying and combining a wide range of data to develop more health-specific indicators. For example, importance of variables such as coping capacity and, critically, political will and governance could be considered in future attempts at mapping vulnerabilities [21,36]. This is essential, although some regions might have high levels of social and economic capital, leading to good scores on the index of adaptive capacity presented here, there is still much adaptation and preparedness work that needs to be done to translate adaptive capacity into practice. To borrow an analogy from thermodynamics, adaptive capacity can be seen as potential energy, which needs political will, commitment, and robust public health and health care institutions to translate it into the kinetic energy required to develop resilient health systems adapted to climate change.

There are in fact myriad relevant health and socioeconomic datasets available that could be amenable as indicators for more health-specific definitions of vulnerability—the issue is that very little work has been done to evaluate their sensitivity and usefulness under different socioeconomic development pathways [34]. The work in this study supports such an observation. Recall that potential proxy indicators such as number of hospital beds or number of medical personnel per capita were unsuitable for use as indicators because of a range of limitations. An additional challenge related to the available data was the varying spatial resolution of datasets; some socioeconomic data are available only at the national level, whereas other data are available at regional levels. National data creates issues because it necessarily disguises sub-national variation and precludes identification of the most and the least vulnerable regions in a country.

Yet the situation appears to be improving. A new generation of climate scenarios, which were not available during the undertaking of this study, is becoming available to the scientific community, and these explore a broader range of socioeconomic variables in modelling the risks of climate change [33]. These scenarios will replace the IPCC SRES scenarios and enable more detailed projections of impacts and vulnerabilities taking into consideration the interactions between climate change and other important disease drivers, such as urbanisation, population settlements, and demographic changes [34]. Furthering developing the health aspects of these scenarios should be a priority research area before applying the new scenarios to develop refined vulnerability indices.

More generally, there is the need to systematically collect and analyse the interlinkages between the numerous and ever-expanding environmental, socioeconomic, demographic and epidemiologic datasets so as to promote the public health capacity to detect, forecast, and prepare for the health threats due to climate change. Health sector engagement with the new climate scenarios will be crucial for achieving this aim [37]. So will efforts to foster data and information exchange, such as the European Environment and Epidemiological (E3) Network developed by the European Centre for Disease Prevention and Control [38], one of many necessary steps towards reducing emerging vulnerabilities due to climate change.

## Supplementary Files

Supplementary File 1 Supplementary Information (PDF, 97 KB)
