# Supplementary material for: Indicators for Tracking European Vulnerabilities to the Risks of Infectious Disease Transmission due to Climate Change
Source: Int J Environ Res Public Health. 2014 Feb 21;11(2):2218–35. doi: 10.3390/ijerph110202218 (PMC3945594; doi:10.3390/ijerph110202218)
Supplement: Supplementary File 1 — Supplementary Information (PDF, 97 KB) [file ijerph-11-02218-s001.pdf]

*Supplementary Information*

# Indicators for Tracking European Vulnerabilities to the Risks of Infectious Disease Transmission due to Climate Change

**Table S1.** Conceptual framework for adaptive capacity.

| Aggregate Dimension of Adaptive Capacity from ESPON [1] | Fit with Variables Identified in [2] | Fit with Variables Identified in [3] |
|---|---|---|
| Awareness | literacy rate among 15–25 year olds<br>literacy ratio (female to male) | high access to information |
| Ability | Population with access to sanitation<br>caloric intake<br>life expectancy at birth<br>maternal mortality | universal health care coverage |
| Action | voice and accountability<br>civil liberties<br>political rights | *per capita* income<br>inequality in the distribution of income |

## References


1. Few, R. Health and climatic hazards: Framing social research on vulnerability, response and adaptation. *Glob. Environ. Change* **2007**, *17*, 281–295.
2. McMichael, A.J. Globalization, climate change, and human health. *N. Engl. J. Med.* **2013**, *368*, 1335–1343.
3. Bowen, K.J.; Ebi, K.; Friel, S.; McMichael, A.J. A multi-layered governance framework for incorporating social science insights into adapting to the health impacts of climate change. *Glob. Health Action* **2013**, *6*, doi:10.3402/gha.v6i0.21820.



© 2014 by the authors; licensee MDPI, Basel, Switzerland. This article is an open access article distributed under the terms and conditions of the Creative Commons Attribution license (http://creativecommons.org/licenses/by/3.0/).
